# Target genes associated with lipid and glucose metabolism in non-alcoholic fatty liver disease

**DOI:** 10.1186/s12944-019-1154-9

**Published:** 2019-12-05

**Authors:** Ting Li, Hua Yan, Yan Geng, Haitao Shi, Hong Li, Shenhao Wang, Yatao Wang, Jingyuan Xu, Gang Zhao, Xiaolan Lu

**Affiliations:** 1 Health Science Center, Xi’an Jiaotong University, NO.76 Yanta West Road, Xi’an, 710061 China; 2 Department of Geratology, Shaanxi Provincal People’s Hospital, Third Affiliated Hospital of Xi’an Jiaotong University, Xi’an, 710068 China; 3 Department of Paediatrics, The Second Affiliated Hospital of Xi’an Jiaotong University, Xi’an, 710004 China; 4 Department of Gastroenterology, The Second Affiliated Hospital of Xi’an Jiaotong University, NO.157 West 5th Road, Xi’an, 710004 China; 5 Department of Gastroenterology, Shanghai Pudong Hospital, Fudan University Pudong Medical Center, 2800 Gongwei Road, Pudong, Shanghai, 201399 China

**Keywords:** NAFLD, Target genes, Lipids metabolism, Glucose metabolism, Gene microarray

## Abstract

**Background:**

Insulin resistance (IR) and lipid peroxidation are accepted as ‘two-hit’ hypothesis of Non-alcoholic fatty liver disease (NAFLD). However, there are few published research on identifying genes which connect lipid and glucose metabolism by gene microarray.

**Objective:**

To identify target genes related to lipid and glucose metabolism that might be responsible for the pathogenesis of NAFLD.

**Methods:**

A rat model of NAFLD was established by feeding male rats with high-fat diet and gene expression profiles of liver tissues were determined using Agilent DNA microarray. We then investigated differentially expressed genes (DEGs) and intersection of them by using Gene Ontology (GO) and Pathway Analyses. Target genes were verified by Real-time polymerase chain reaction (RT-PCR).

**Results:**

Compared with control, 932 genes, including 783 up-regulated and 149 down-regulated, exhibited differences in expression. The up-regulated genes were involved in biosynthesis, cell development, cell differentiation and down-regulated genes contributed to biological metabolic process, adipokine metabolic pathway and insulin signaling pathway. We identified genes involved in insulin signaling pathway, Notch signaling pathway and lipid synthetic process to be closely related to liver fat accumulation and insulin resistance. Among them, IGFBP7, Notch1 and HMGCR were up-regulated (2.85-fold, 3.22-fold, and 2.06-fold, respectively, all *P* < 0.05) and ACACB was down-regulated (2.08-fold, *P* < 0.01). These four genes supposed to connect lipid and glucose metabolism after GO and Pathway analyses.

**Conclusions:**

These findings provide innovative information on the whole genome expression profile due to high-fat diet feeding, and bring new insight into the regulating effects of genes on the lipid and glucose metabolism of NAFLD.

## Introduction

Non-alcoholic fatty liver disease (NAFLD) belongs to a metabolic stress-induced liver disease [1]. NAFLD is increasingly recognized as the most common chronic liver disease [2] and covers a spectrum of liver disorders ranging from steatosis to steatohepatitis and cirrhosis [3]. Insulin resistance and lipid peroxidation have been widely accepted as the ‘two-hit process’ of NAFLD [4], accumulation of hepatic diacylglycerol impairs insulin receptor activation and glycogen synthesis, insulin resistance in turn increases flux of substrates that promote lipogenesis and gluconeogenesis [5]. However, it remains unclear which biomarkers are responsible for the vicious cycle between lipid and glucose metabolism.

To better understand the impact of diet restrictions on NAFLD murine models have been developed to experimentally mimic human diet caused NAFLD, the combination of metabolic phenotyping with specific gene expressions provides a top-down approach to illuminate the metabolic pathways that are modulated by such restrictions. Recently, researchers have applied microarray analyses to clarify the mechanisms of NAFLD. Johannie et al. observed increased expression of genes that regulate inflammation in adipose tissues from patients with NAFLD and the expression of these genes increased as disease progressed [6]. The activity of lipid metabolism and cell apoptosis in NAFLD were increased with the enhanced expression of plin, acsl6, scd2, etc. [7]. Simon T et al. reported the genetic architecture of diet-induced hepatic fibrosis in mice and found 10 enriched pathways including lipid metabolism (fatty acid metabolic process, oxidation-reduction process), and insulin signaling (PI3K-Akt signaling pathway) [8]. However, there are few published researches on identifying genes which connect lipid and glucose metabolism by gene expression profiles.

In order to screen potential target genes from systematic perspective, we conducted Rat 4x44K gene microarray which covered 19,246 genes to detect liver tissues from male rats following 14 weeks of high-fat diet. In addition, bioinformatics analyses were performed to explore the biological function of DEGs. The data presented could provide useful information on the global gene expression changes and bring new insights into the mechanisms of lipid and glucose metabolism in NAFLD.

## Methods

### Animal studies

Adult 8-week -old healthy male Sprague-Dawley (SD) rats (246.35 ± 18.57 g) were supplied by the Laboratory Animal Center of Xi’an Jiaotong University (Xi’an,China), and the vivarium were kept at a temperature between 20 °C and 24 °C with a 12 h dark/light cycle under specific pathogen-free conditions with free access to water and maintained at the Laboratory Animal Center of Xi’an Jiaotong University with standard monitoring thereafter. A total of 24 rats were randomly divided into two groups: NAFLD vs Normal Control (NC) (*n* = 12 for each group), and fed with different chows for 14 weeks respectively: a high-fat diet (HFD) consisted of: 10% lard, 2% cholesterol, 5% saccharose, 0.5% hog bile extract and 82.5% standard chow diet, laboratory chow consisted of: NC:5% fat, 25% protein, 70% carbohydrate. All experiments and the protocol were approved by the Animal Care Committee of Xi’an Jiaotong University. The body mass was recorded weekly. Blood samples were collected from the tail vein and stored at − 80 °C until biochemical analyses. Rats were sacrificed by an intravenous administration of pentobarbital (overdose) at the end of the experiment. Small cuboids around 5× 5 × 2–3 mm were cut out from the middle part of the right liver lobe and placed into the 10% formalin solution for histopathology, the other liver tissues were collected in RNase-free tubes and stored at − 80 °C until tissue examination. All operations and handling procedures were carried out in accordance with the current Animal Protection Law of China.

### Biochemical assays and histological analyses

Serum fasting blood glucose (FPG), triglyceride (TG), total cholesterol (TC), low density lipoprotein (LDL), high density lipoprotein (HDL), alanine aminotransferase (ALT), aspartate aminotransferase (AST) were measured using an automatic biochemical analyzer. Small cuboids from the right lobe of liver were fixed in 10% buffered formaldehyde, embedded in paraffin and sectioned at 5 μm thickness, then stained with hematoxylin for 5–10 min, immerged in ammonia water for 30 s, and counterstained with 0.5% erosin for 2–5 min. The slices were dehydrated by gradient ethanol, cleared in xylene. Finally, they were sealed with neutral gum. Histopathologic examinations of the liver sections were conducted by a pathologist and peer-reviewed.

### Rat 4x44K gene expression array detection

Total RNA was isolated from the rat liver samples using TRIzol reagent (Invitrogen, Carlsbad, CA, USA) and purified using the RNeasy Mini Qiagen purification kit (QIAGEN, Valencia, CA) according to the manufacturer’s instructions. Then, Total RNA was amplified and transcribed into fluorescent cRNA by using the manufacturer’s Agilent’s Quick Amp Labeling protocol (version 5.7, Agilent Technologies). The labeled cRNAs were hybridized onto the Whole Genome Oligo Array (4x44K, Agilent Technologies). After having washed the slides, the arrays were scanned by the Agilent Scanner G2505C. Agilent Feature Extraction software was used to analyze acquired array images. Quantile normalization and subsequent data processing were performed using the GeneSpring GX v11.5.1 software package (Agilent Technologies). This gene expression array detection was finished by Shanghai Kang Chen Bio-Tech Company.

### Microarray data analyses

Differentially expressed genes between two groups (NC vs NAFLD) with statistical significance were identified through *P* < 0.05 and Fold Change ≥2.0 after filtering the raw data. For example, the gene with ≥ two-fold higher expression than the NC group was regarded as up-regulation; the gene with ≥ two-fold lower expression than the NC group as down-regulation. Then, Hierarchical clustering analysis was used to arrange samples into groups based on their expression levels, which helps to illustrate relations among samples. GO analysis was used to associate DEGs with GO categories (www.geneontology.org). Pathway analysis is a functional analysis mapping genes to KEGG pathways. After combining GO and Pathway analyses, the glucose and lipid metabolism related genes were screened.

### Quantitative real-time PCR

To verify the gene chip data, four glucose and lipid metabolism-related genes were selected for qRT-PCR analyses. Primer sequences were designed by Primer Express 2.0 software according to mRNA sequences of four target genes HMGCR, IGFBP7, Notch1, ACACB and internal control GAPDH. They were synthesized by Beijing AuGCT DNA-SYN Biotechnology(Table 1). cDNA was synthesized from the total RNA using a PrimeScript®RT reagent kit (Takara, Dalian, China) and primers. The qRT-PCR reactions were performed on a Bio-Rad iQ5 real-time thermal cycler using a SYBR® Premix Ex Taq™II kit (Takara) in a 20 μl reaction system. All of the PCR cycling conditions were modified to 95 °C for 30s, followed by 40 cycles of 95 °C for 5 s, 60 °C for 20s, and 65 °C for 15 s. All reactions were performed in triplicate, and the results were represented as relative mRNA expression data which were calculated using the 2^-∆∆CT^ method.

**Table 1:** primer sequences used in qRT-PCR

Genes	Accession numbers	Primer sequences(5’to3’)
HMGCR	NM_013134	^a^FP:5′-CAATGGCAACAACAGAAGG-3′
		^b^RP:5′-CACAAGCACGAGGAAGAC-3’
IGFBP7	NM_001013048	FP:5′-TCACCCAGGTCAGCAAAG-3’
		RP:5′-GTCACCAGGCAAGAGTTC-3’
Notch1	NM_001105721	FP:5′-GGTGGACATTGACGAGTG-3’
		RP:5′-GGCATAAGCAGAGGTAGTAG-3’
ACACB	NM_053922	FP:5′-CGGCTACTACCTGGACATC-3’
		RP:5′-TGGAATCGCTTGGCTTGG-3’
GAPDH	NM_017008	FP:5′-ATGGTGAAGGTCGGTGTGAACG-3’
		RP:5′-CGCTCCTGGAAGATGGTGATGG-3’

^a^FP: forward primer

^b^RP: reverse primer

### Statistical analysis

Data were analyzed using SPSS 19.0. The Student’s t-test or Mann-Whitney U test were conducted for data analyses when appropriate. Results were expressed by the means ± SEM. A *p*-value ≤0.05 was considered statistically significant.

## Results

### Body mass, biochemical and histopathological alterations in NAFLD and control profiles

**Fig. 1:**
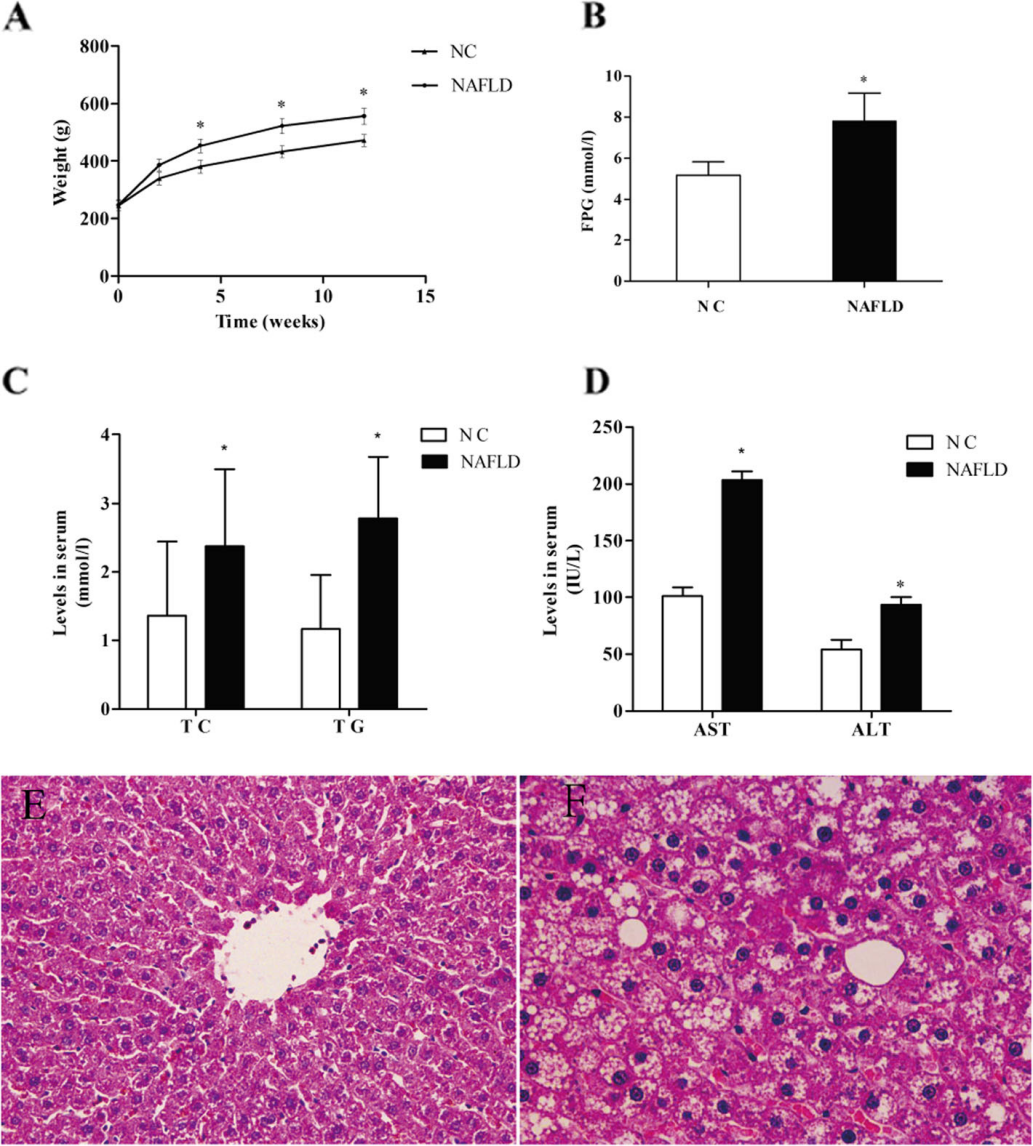
(**a**) Body weight were measured using weighing scale; (**b**) Fasting plasma glucose and (**c**) total TC, TG and (**d**) ALT, AST were measured using an automatic biochemical analyzer. All data were expressed as the mean ± SEM.^*^P < 0.05; Compared to the normal control group. Changes in the rat livers after 14 weeks of treatment.H&E staining of paraffin-embedded sections of rat livers. (**e**) Histopathological changes of the liver tissues obtained from normal control rats. (**f**) Histopathological changes of the liver tissues of NAFLD rats (Original magnification, × 400)

There were no premature deaths during the experiments. Following high-fat diet exposure, rats experienced an increase in body mass (*P* < 0.05) (Fig. 1a) than the other’s compared with the control group animals. Significant and substantial changes of the mass of NAFLD rats were observed between the fourth and eighth week. Similarly, we observed that the serum concentrations of ALT, AST, FPG, TC, TG in NAFLD group were higher than that of NC group animals (*P* < 0.05) (Fig. 1b-d).Interestingly, for the normal control group, the structure of hepatic lobes was clear, and hepatocytes were orderly arranged in cords radiation from the central vein by using H&E staining. No significant lipid droplets or lobular inflammatory cell infiltration were observed (Fig. 1e) throughout the tome course study. In contrast, there were typical vesicular steatosis, lipid droplets and hepatocellular ballooning in the NAFLD group (Fig. 1f).

### Global gene expression profiles in liver tissues of NAFLD and control rats

Total 23,381 annotated and unannotated genes were detected with the use of Agilent DNA microarray. 932 differentially expressed genes were screened after filtering the raw data according to *p* < 0.05 and fold change ≥2.0. Among them, up-regulations of 783 genes and down-regulations of 149 genes were significantly observed in mice compared with the control. In the meantime, we conducted hierarchical clustering to group DEGs based on similarities of expression patterns shown in Fig. 3a. Similar to the tendency of changes were observed.

### Gene ontology analysis of differentially expressed genes

**Fig. 2:**
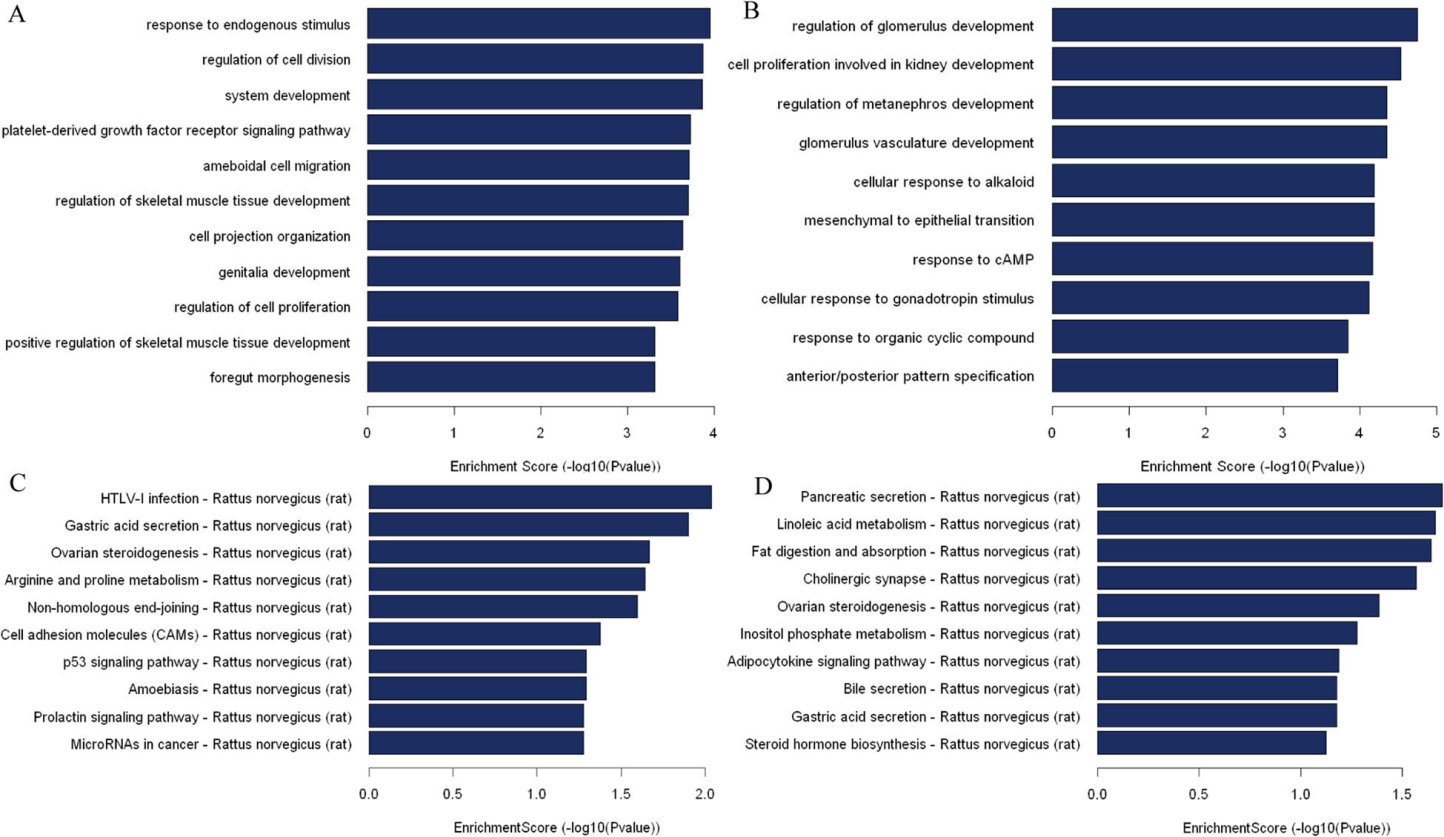
GO and Pathway analyses were applied to explore the differntially expressed genes. GO analyses: (**a**) Biological process classification of up-regulated genes. (**b**) Biological process classification of down-regulated genes. Pathway analyses: the bar plot shows the top ten Enrichment score value of the significant enrichment pathway. (**c**) Pathway associated with up-regulated genes. (**d**) Pathway associated with down-regulated genes

The biological function of 932 differentially expressed genes were further studied by GO analysis. The bar plot was indicative of the top ten Enrichment Score value of the significant enrichment terms, and the up-regulated genes were mainly involved in the biological process of endogenous stimulus (Notch1, IGFBP7, GCK, INS1), regulation of cell division (PDGFB, SOX17), system development (Notch1, CCL14, HMGCR, IGFBP7, ESR2, BMP6), and cell proliferation (Notch1, HMGCR, HYAL1, BMP6, SOX17) (Fig. 2a). The down-regulated genes were mainly involved in the response to organic cyclic compound (ACACB, EGR1, CRHBP) and regulation of glomerulus development (EGR1, BMP4, WT1) (Fig. 2b). All of the above results indicated that expressions shifts of genes were significant across all treated mice. Additionally, glucose and lipid metabolism related biological processes were further screened by GO analysis in this work, including regulation of gluconeogenesis (GCK, NR3C1), insulin secretion process (TCF7L2, HMGCR. GCK), glucose homeostasis (GCK, TCF7L2, HMGCR), regulation of metabolic process (ESR2, NOTCH1, ID3), lipid biosynthetic process (HMGCR, IGFBP7, ALDH1A1), lipoprotein metabolic process (ATM, HHATL), steroid biosynthetic process (NR3C1, BMP6, IGFBP7), organic acid biosynthetic process (ACACB), and carboxylic acid metabolic process (ACACB, FABP2, CYP17A1).

### Pathway analysis of differentially expressed genes

KEGG pathway analysis was also used to investigate the differentially expressed genes. The constructed bar plot showed the top ten Enrichment Score value of the significant enrichment pathway to compare metabolic pathway profiles of mice. The up-regulated genes were mainly involved in gastric acid secretion pathway (ADCY1, MYLK3), arginine and proline metabolic pathway (CKM, GLUL, LAP3), ovarian steroidogenesis pathway (ADCY1, BMP6, INS1) (Fig. 2c). The down-regulated genes mainly contributed to pancreatic secretion pathway (ADCY9, PLA2G2D), linoleic acid metabolism pathway (CYP2C24, PLA2G2D), fat digestion and absorption pathway (FABP2, PLA2G2D), adipokine metabolic pathway (ACACB, IRS3) and bile secretion pathway (ADCY9, SLC4A4) (Fig. 2d). We further screened the lipid and glucose metabolism related pathways, including Glycolysis related pathway (ADH6, GCK), insulin secretion pathway (ADCY1, GCK, INS1), insulin signaling pathway (ACACB, IRS3), fat digestion and absorption pathway (FABP2, PLA2G2D), bile secretion pathway (ADCY9, SLC4A4, HMGCR), and adipokine metabolic pathway (ACACB, IRS3). The metabolic pathway alterations induced by NAFLD compared with the control are shown in Fig. 2c and are summarized for the pathway shifts of each metabolite.

### Screening of candidate genes related to glucose and lipid metabolism

**Fig. 3:**
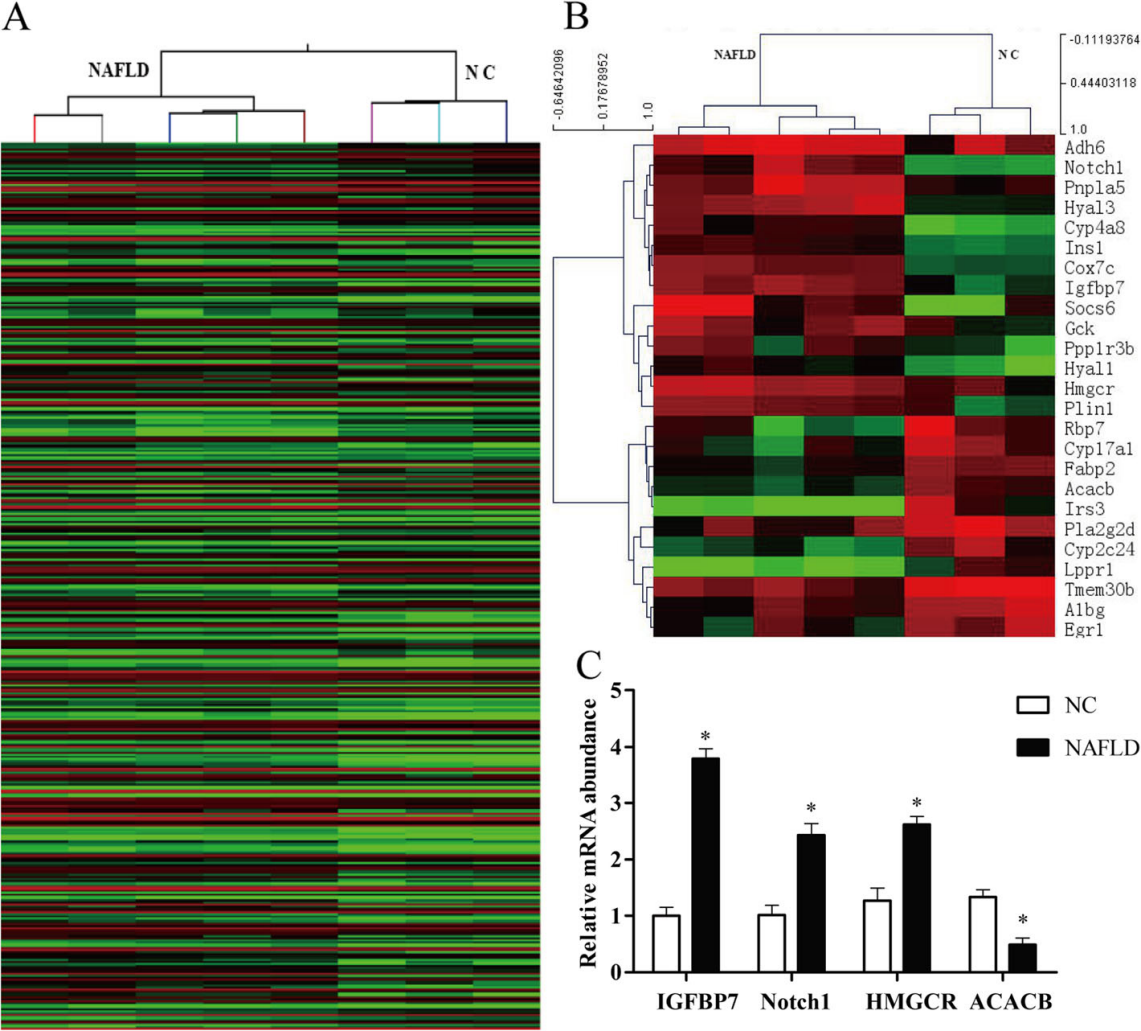
(**a**) Hierarchical clustering analysis of differentially expressed genes in NAFLD and NC group. Red and green colors denote the expression level higher and lower than the control group. (**b**) Hierarchical clustering analysis of glucose and lipid metabolism-related genes in liver tissues. (**c**) Validation of gene chip results by real-time PCR (*P < 0.05)

A total of 25 genes were screened as the candidate genes, including 14 up-regulated and 11 down-regulated (Tables 2 and 3) with the use of GO and pathway analyses. Consistent with the above observations, the glucose and lipid metabolism related genes in NAFLD group were distinctly different from NC group after Hierarchical clustering analysis (Fig. 3b). Among them, IGFBP7 was increased 2.85-fold (*P*-value 0.0005) while Notch1 was increased 3.22-fold (*P*-value 0.0034) compared to control. HMGCR which contributed to insulin secretion and lipid biosynthetic process was also increased 2.06-fold (*P*-value 0.0106). ACACB which involved in insulin signaling pathway and adipokine metabolic pathway was decreased 2.08-fold (*P*-value 0.0021) compared to control. These four genes supposed to connect glucose and lipid metabolism were selected and validated by real-time PCR.

**Table 2:** The up regulated genes in lipid and glucose metabolism

Gene Symbol	Fold Change	*P*-value	Accession numbers
HMGCR	2.0603	0.0106	NM_013134
ADH6	2.0391	0.0487	NM_001012084
CYP4A8	3.1672	0.0001	NM_031605
COX7C	2.8442	0.0052	NM_001134705
PLIN1	2.3422	0.0066	NM_013094
PNPLA5	2.2352	0.0170	NM_001130497
IGFBP7	2.8527	0.0005	NM_001013048
Gck	2.2979	0.0477	NM_001270849
INS1	2.0864	0.00007	NM_019129
Ppp1r3b	2.0870	0.0459	NM_138912
Hyal1	2.7724	0.0138	NM_207616
SOCS6	4.3454	0.0330	NM_001271149
Hyal 3	3.1806	0.0002	NM_207599
NOTCH1	3.2204	0.0034	NM_001105721

**Table 3:** The down regulated genes in lipid and glucose metabolism

Gene Symbol	Fold Change	*P*-value	Accession numbers
ACACB	2.0838	0.0021	NM_053922
PLA2G2D	2.3940	0.0203	NM_001013428
FABP2	2.1024	0.0006	NM_013068
LPPR1	3.5070	0.0031	NM_201271
Cyp17A1	2.5035	0.0222	NM_012753
Cyp2C24	2.7279	0.0062	NM_001271354
Tmem30b	2.9709	0.0050	NM_001080380
IRS3	4.7067	0.0045	NM_032074
A1BG	2.3833	0.0037	NM_022258
RBP7	2.6895	0.0385	NM_001108693
EGR1	2.4003	0.0166	NM_012551

### Validation of gene chip results by real-time PCR

Real-time PCR was used to detect the expression changes of four genes (IGFBP7, Notch1, HMGCR and ACACB) in liver tissues. The results showed that the expression of IGFBP7, HMGCR and Notch1 were increased (*P* < 0.05) and expression of ACACB was down-regulated (*P* < 0.05) compared with NC group. The data from real-time PCR analysis had similar gene expression trend with gene microarray (Fig. 3c).

## Discussion

Dietary habits are closely related to the occurrence and development of diseases, especially in metabolic syndrome. Scicchitano P et al. provided an overview that nutraceuticals and functional food ingredients effectively reduced the cardiovascular risk induced by dyslipidaemia, the mechanisms may be related to decrease the expression of 7α-hydroxylase and 3-hydroxy-3-methylglutaryl-CoA reductase (HMGCR) [9]. Conversely, modeling metabolic syndrome is commonly accomplished with the use of chow diets enriched with carbohydrates and/or lipids. Central obesity, hyperglycemia, and earlier onset of the metabolic aberrations and additionally, dyslipidaemia can be induced by High-fat diets. NAFLD was observed in the rats those received HFD the but not in those that were deprived of some other nourish elements. Our results demonstrated that dietary intake, particularly of long-term high fat and sugar content can gradually affected hepatic lipid and glucose metabolism which led to the over accumulation of lipid in the hepatic cells and insulin resistance. However, the mechanisms underlying such actions are not fully understood. Recently, Gene microarray has become effective way to investigate pathogenesis of NAFLD [10]. Previous studies have found inflammation response, oxygen stress and carbohydrate metabolism were closely related to NAFLD [11]. We used Rat 4x44K gene expression array to detect gene profiles of liver tissues from the male rats following 14 weeks of high-fat diet, we characterized the metabolic perturbations and metabolic pathway profiles alterations induced by NAFLD in the rat and found biological metabolism including regulation of gluconeogenesis, lipid biosynthetic process were over-represented and genes associated with pancreatic secretion pathway, adipokine metabolic pathway and insulin signaling pathway were down-regulated compared to NC group. It could explain that chronic high-fat diet caused dysregulation expression of lipid and glucose metabolism related genes which led to increased concentrations of TG content, serum cholesterol and Fasting Plasma glucose (FPG).

Insulin resistance is the primary pathogenesis of NAFLD. NAFLD often impacts glucose and lipid metabolism by exacerbating hepatic IR [12]. Genes in insulin secretion, insulin signaling pathway and Glycolysis/Gluconeogenesis pathway were differentially expressed compared to NC group. The SOCS protein family could contribute to insulin resistance [13], suppressor of cytokine signaling 6(SOCS6) was up-regulated and involved in the intracellular signal transduction pathway with Glucokinase (GCK). As a main factor in glycolysis or gluconeogenesis in vivo [14], GCK was also up-regulated and supposed to be compensatory effects on insulin resistance. Dysfunction of IRS-1 and IRS-2 would lead to insulin resistance and NAFLD [15]. The recovery of insulin signaling pathway mainly depended on insulin receptor substrate 3 (IRS3) phosphorylation in IRS-1 knockout mice [16]. IRS-3 was down-regulated and involved in insulin signaling pathway, implying IRS-3 may induce insulin resistance of NAFLD.

Long-term high fat exposure caused alterations in lipid and cholesterol metabolism genes which contributed to the impaired processes of lipid intake, synthesis and transport [17]. CYP4A was regulated by PPARα in the lipid metabolism of nonalcoholic steatohepatitis [18], Cyp4A8 was up-regulated and high expression of CYP4A could decompose fatty acids into dicarboxylic fatty acids which damage mitochondrion function and generated more ROS [19], this may explain why fatty acid degradation pathway which Cyp4A8 involved in was over represented**.** A greater fatty acids availability may favor Cyp4A8 that preferentially use lipids. In addition, the increasing needs for dietary fats can further induce the lipid synthesis process and then reduce the decomposition of other nutrients. As the gene of lipid droplet coat protein, PLIN1, which may induce lipase LIPE to transport from the cytoplasm into lipid drops [20], was also up-regulated, consistent with previous study [7]. Multiple genes within the biological process of lipid biosynthetic process, including HMGCR, insulin-like growth factor binding protein 7 (IGFBP7), platelet-derived growth factor beta polypeptide (PDGFB), bone morphogenetic protein 6 (BMP6) were up-regulated, inferring synthesis of lipid was increased during the occurrence of NAFLD.

**Fig. 4:**
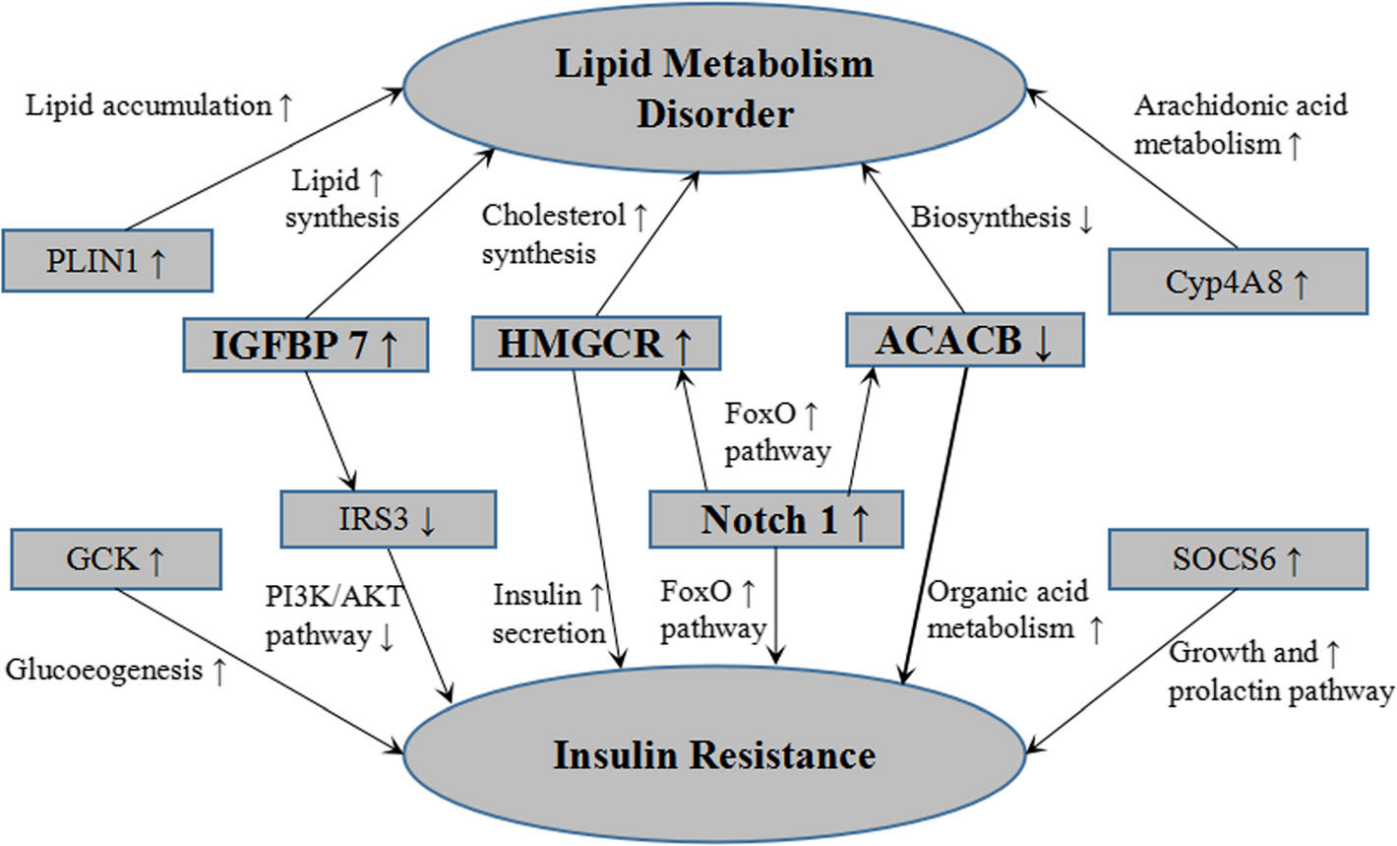
The connections of genes related to glucose and lipid metabolism in NAFLD

In the present work, another interesting finding was explored, IGFBP7, HMGCR, Notch1 and ACACB were supposed to connect glucose and lipid metabolism after GO and pathway analyses (Fig. 4). IGFBP7 was a high-affinity insulin binding protein which blocked insulin binding to its receptor [21], and inhibited expression of IRS-1 and IRS-2 [22]. We noted that IGFBP7 was up-regulated while IRS-3 was down-regulated and involved in insulin signaling pathway, implying that IGFBP7 may induce insulin resistance by inhibiting IRS-3. IGFBP7 also involved in lipid and steroid synthesis, suggesting it could increase lipogenesis of NAFLD. Consistent with this involvement, as a target gene of SREBP-2 in cholesterol synthesis process [23], HMGCR was up-regulated and contributed to lipid synthesis with IGFBP7. We found HMGCR also involved in insulin secretion and glucose homeostasis, previous study suggested that the increased risk of type2 diabetes noted with statins was partially explained by HMGCR inhibition [24], inferring that HMGCR could be the potential medium between the glucose and lipid metabolism in NAFLD.

Notch signaling pathway was distinctly different with NC group and has a notable impact on the lipid and glucose metabolism. Notch1 was suppressed via curcumin treatment, which in turn ameliorated fatty liver and insulin resistance [25]. In this study, Notch1 was up-regulated while FoxO pathway was over-represented, Notch1 could up-regulate FoXO1 which aggravated insulin resistance of NAFLD [26]. This result was previously shown to be due to up-regulated FoXO1. Thus, increased expression of FoXO1 may be a reason of aggravated insulin resistance. In addition, Notch pathway inhibited ACACB which led to IR along with a steatotic liver [27]. ACACB inhibited fatty acid beta oxidation in the normal circumstances, expression of ACACB decreased in obesity and diabetes mice with the increase of time [28]. Lower excretion of derivatives, which are intermediate of fatty acid beta oxidation, indicated a down regulation of lipids oxidation despite having contained the same amount of fat. In ACACB knockout rat, PPARα, SREBP-1 and SREBP-2 were up-regulated to compensate the increased FFAs consumption [29]. It was observed that ACACB was down-regulated and involved in moderate perturbations in the insulin signaling pathway, adipokine metabolic pathway and organic acid metabolism. Implying that down regulation of ACACB could reduce hepatic fat content, which in turn would improve hepatic insulin sensitivity in NAFLD due to increased hepatic mitochondrial oxidation and reduced de novo lipogenesis.

## Conclusions

In conclusion, this study analyzed the expression profiles of liver tissues of NAFLD rats following 14 weeks of high-fat diet and found varieties of physiological activities were implicated in NAFLD, especially the lipid and glucose metabolism. In addition, after GO and Pathway analyses, Four genes (IGFBP7, Notch1, HMGCR and ACACB) which were involved in both glucose and lipid metabolism were screened out, and they were supposed to associate with the vicious cycle between glycolipid metabolism which may aggravate the progression of NAFLD. Therefore, additional studies will be needed to elucidate the regulating effects of these four genes in NAFLD.

## Data Availability

The data will be available on request.

## Abbreviations

ACACB: Acetyl-coA carboxylase beta
ALT: Alanine aminotransferase
AST: Aspartate aminotransferase
DEGs: Differentially expressed genes
FPG: Fasting blood glucose
GCK: Glucokinase
GO: Gene ontology
HDL: High density lipoprotein
HMGCR: 3-hydroxy-3-methylglutaryl coenzyme A reductase
IGFBP7: Insulin-like growth factor binding protein7
IR: Insulin resistance
IRS3: Insulin receptor substrate3
LDL: Low density lipoprotein
NAFLD: Non-alcoholic fatty liver disease
NC: Normal control
RT-PCR: Real-time polymerase chain reaction
TC: Total cholesterol
TG: Triglyceride
